# The prevalence of trachoma, ocular *Chlamydia trachomatis* infection and anti-Pgp3 antibodies in Choiseul Province, Solomon Islands

**DOI:** 10.1371/journal.pntd.0013381

**Published:** 2025-09-08

**Authors:** Clare E. F. Dyer, Carleigh S. Cowling, Oliver Sokana, Lazarus Neko, Nemia Bainivalu, Freda Pitakaka, Anasaini Cama, Mitchel Starr, Emma M. Harding-Esch, Cristina Jimenez, Ana Bakhtiari, Sarah Boyd, Sara Webster, Anthony W. Solomon, John M. Kaldor, Susana Vaz Nery

**Affiliations:** 1 The Kirby Institute, University of New South Wales, Sydney, Australia; 2 Ministry of Health and Medical Services, Honiara, Solomon Islands; 3 The Fred Hollows Foundation, Melbourne, Australia; 4 New South Wales State Reference Laboratory for HIV, St Vincent’s Centre for Applied Medical Research, St Vincent’s Hospital, Sydney, Australia; 5 London School of Hygiene & Tropical Medicine, London, United Kingdom; 6 Sightsavers, Haywards Heath, United Kingdom; 7 International Trachoma Initiative, The Task Force for Global Health, DecaturUnited States of America; 8 Global Neglected Tropical Diseases Programme, World Health Organization, Geneva, Switzerland; The University of Kansas, UNITED STATES OF AMERICA

## Abstract

Trachoma, caused by repeated ocular infection with *Chlamydia trachomatis*, remains a leading infectious cause of blindness globally, with significant implications for public health. The World Health Organization and partners aim to eliminate trachoma as a public health problem by 2030, targeting specific prevalence thresholds for trachomatous trichiasis (TT) and trachomatous inflammation—follicular (TF). Diagnosis is primarily clinical. Studies have shown discrepancies between prevalence estimates of TF and *C. trachomatis* infection. This study, undertaken in Choiseul, Solomon Islands, evaluated TF, evidence of current *C. trachomatis* infection (by polymerase chain reaction (PCR) on conjunctival swabs), and evidence of past exposure to that bacterium (using anti-Pgp3 serology on dried blood spots). Among 645 1–9-year-old children, TF prevalence was 17.5% and *C. trachomatis* prevalence was 8.5%. These findings suggest transmission of sufficient intensity to pose a public health problem. Notably, 59% of children with TF had evidence of neither current nor previous *C. trachomatis* infection. Increasing age was associated with TF and evidence of past infection, but not current infection. The community had poor water, sanitation, and hygiene conditions. This study highlights the benefit of integrating laboratory testing for guiding effective trachoma elimination as a public health problem. Although our work was limited by imperfect enrolment of resident children and the logistical challenges of collecting samples in a remote region, we believe our data justify continued public health interventions against trachoma in Choiseul.

## Author summary

This study looked at the signs of trachoma, the world’s leading infectious cause of blindness, in children in the Solomon Island province of Choiseul, exploring evidence of past and current *C. trachomatis* infection*.* We show that trachoma is a public health problem in Choiseul and can be attributed to *C. trachomatis.* Public health interventions are required, including antibiotic mass drug administration and improvements in access to clean water and sanitation. As we pursue the global elimination of trachoma, this survey shows that integrating laboratory testing for current and previous *C. trachomatis* infection is feasible and a useful exercise.

## Introduction

Trachoma is the leading infectious cause of blindness globally, responsible for almost 2 million cases of blindness and visual impairment. Caused by an inflammatory response to repeated ocular infections with *Chlamydia trachomatis*, over time the eyelid develops scarring (“trachomatous scarring”, TS), which can cause eyelashes to turn inwards so that they touch the eyeball (“trachomatous trichiasis”, TT) precipitating corneal opacity (CO) and potentially irreversible blindness [1]. World Health Organization (WHO) Member States have set a global target for elimination of trachoma as a public health problem by 2030 [2]. Elimination of trachoma as a public health problem is defined as (i) a prevalence of TT unknown to the health system of less than 0.2% in individuals aged 15 years and older in each formerly endemic district; (ii) a prevalence of trachomatous inflammation—follicular (TF) of less than 5% in children aged 1–9 years in each formerly endemic district; and (iii) evidence that the health system can identify and manage incident cases of TT [2].

WHO and partners promote the “SAFE” (Surgery, Antibiotic mass drug administration [MDA], Facial cleanliness, Environmental improvement) strategy for trachoma’s elimination as a public health problem: S for TT; and AFE to clear *C. trachomatis* infection and reduce its transmission [2]. The threshold for initiating the AFE components of the strategy is a TF prevalence of 5% or greater in children aged 1–9 years. The threshold for initiating the S component of the strategy at population level is a TT prevalence of 0.2% or greater in individuals aged 15 years and older [3].

The first evidence of *C. trachomatis* infection is the presence of mild inflammation in the conjunctiva, followed by the development of a follicular response that may meet the criteria for the sign of TF. In some cases, more severe inflammation may be observed, perhaps sufficient to qualify as trachomatous inflammation—intense (TI). TF and TI are typically found predominantly in younger children, since infection spreads more easily in this age group [1,2,4].

The WHO simplified clinical trachoma grading system [1] defines the presence of TF and/or TI as “active trachoma”, with the presumption being that these signs are caused by *C. trachomatis* infection [2,5]. The development of molecular techniques, namely the use of polymerase chain reaction (PCR) on conjunctival swabs, to detect bacterial DNA and therefore confirm infection, has shown that TF is a poor predictor for the presence of ocular *C. trachomatis* infection [6,7], especially post-MDA [8], with several populations demonstrated to have moderate to high TF prevalence and limited to no *C. trachomatis* infection [9,10]. On the other hand, given that bacterial DNA may be present in the eye before signs like TF appear and that PCR has high sensitivity, some other studies have shown a higher prevalence of *C. trachomatis* infection than TF [11,12]. Recently, there has also been the development of serological approaches that detect anti-Pgp3 antibodies and provide robust data for evaluating population-level exposure to *C. trachomatis* and therefore a convenient, non-invasive method to monitor trachoma transmission intensity, given they can be used on dried blood spots (DBS) through a finger-prick [13]. Anti-Pgp3 IgG antibodies demonstrate durable persistence with a median half-life ranging from 3.0 to 5.7 years in endemic settings in Ethiopia and Tanzania, characterized by low seroreversion rates ranging from 0 to 2.5 per 100 person-years, supporting their utility as reliable markers for assessing *C. trachomatis* exposure in trachoma control programs [11,14]. An enzyme-linked immunosorbent assay (ELISA) and lateral flow assay (LFA) detecting anti-Pgp3 antibodies are valuable serosurveillance tools. Reagents for both assays were developed by the Centers for Diseases Control and Prevention (CDC), Atlanta GA, USA. ELISA is considered a robust test but requires specialised laboratory equipment and statistical analysis to determine positive vs negative results. On the other hand, LFA offers a technically simpler option that can be easily deployed in low resource settings [15–17].

Choiseul is one of nine provinces of the Solomon Islands and has been defined as an evaluation unit for trachoma programme work. Baseline trachoma mapping was conducted from 2012–2013 using the standard protocols of the Global Trachoma Mapping Project (GTMP) [18], with technical support for surveys now provided by Tropical Data (TD) [19]. The baseline TF prevalence in 1–9-year-olds was 6.1% [20]. Antibiotic MDA was conducted in 2015, with 96% coverage. In September 2016, 12 months after MDA, an impact survey was conducted with TD support. This showed a TF prevalence of 2.2%, and a TT prevalence of 0%, indicating elimination thresholds of TF and TT had been achieved and allowing a period of surveillance to begin. In October 2019, a surveillance study was conducted to again estimate trachoma prevalence. Unexpectedly, the prevalence of TF had increased to 10.6%, while the TT prevalence was still 0% (S1 Table). Given that TF was now above the 5% WHO threshold for MDA there was a need to investigate whether the follicular conjunctivitis observed in Choiseul could be attributed to current or previous *C. trachomatis* infection and if trachoma was a public health problem requiring additional MDA in this province. We approached this through the collection of conjunctival swabs for PCR and DBS for anti-*C. trachomatis* serology (by both ELISA and LFA), alongside examination for TF. Our results provide evidence to inform the Solomon Islands trachoma elimination program on the need for further MDA and scaling up of F&E interventions in Choiseul.

## Methods

### Survey design

Fifteen villages (clusters) were selected using the five villages that had the highest cluster-level TF prevalences in the 2019 surveillance survey (22.5–50%), which we labelled as “index” villages, plus two neighbouring villages per index village.

In each selected village, both index and neighbouring, all children aged 1–9 years were invited to participate, corresponding to a total of 1,300 children in the 15 villages.

Data on household GPS coordinates, household-level water, sanitation and hygiene (WASH) variables, as well as clinical trachoma grading from children aged 1–9 years were captured in the TD app on an Android mobile phone, as per the usual TD methods which have been previously described [19]. In addition, barcode information was captured to link the conjunctival swab and DBS samples to the participant for analysis purposes. Information about the research project, including the purpose, aims, and requirements, was provided to participants and their parent(s) or guardian(s), using a culturally appropriate flip chart as well as a hardcopy participant information statement. Written consent was then obtained from a parent or guardian for the collection of conjunctival swabs and DBS for each participant. Verbal consent was sought from the child for ocular examination.

### Field procedures and data collection

Fieldwork was conducted in November–December 2020 by one team of TD-trained personnel. The team consisted of a TF grader who was also the team leader, a sample manager and a data recorder. Flipcharts, consent forms and other tools can be made available on request. A structured questionnaire [19] was undertaken by the head of the household, covering WASH and household variables, including number of household residents and information regarding sanitation and water sources. Household latrine and handwashing facilities, if present, were inspected to verify details of their availability and type [19].

Participants were examined by a TD-certified grader for signs of trachoma using the WHO simplified grading system [1]. Standard procedures, using 2.5 × binocular magnifying loupes (OptiVISOR LX, Donegan Optical Company), sunlight or a torch for illumination, and follicle size guides to aid TF diagnosis were used [21]. Data on TT, TF and TI were recorded.

After examination, a polyester-coated cotton swab was passed three times over the everted left conjunctiva (the second eye to be examined) with a 120-degree turn between each pass. The swab was placed into a PCR transport tube (Cobas, Roche Diagnostics).

In addition, one of the participant’s fingers was cleaned with alcohol and then pricked using a single use, sterile lancet, with up to 5 drops of blood collected onto Whatman 903 protein saver card (Thermofisher Scientific) and air-dried for a minimum of 4 hours. Once dry, individual DBS cards were placed in a Whatman foil barrier bag (Bio-strategy) containing two 1g silica gel desiccants (Desicco Pty Ltd). Conjunctival swabs and DBS were stored at ambient temperature in the field and kept in a refrigerator (approx. 4°C) in Honiara for approximately two weeks before being shipped. Conjunctival swabs and DBS were then shipped to St. Vincent’s Centre for Applied Medical Research, Sydney, Australia at room temperature and stored at 2–8°C and -20°C respectively, until testing.

Gloves were used by the staff member collecting the DBS and conjunctival swab and changed between each participant. Alcohol gel was used to clean team members’ hands between each participant. Following WHO recommendations, children were held by a parent or assistant recruited from the local population (often the village leader) to help them keep still during the examination and sample collection [22].

Each study participant was allocated a unique 5-digit barcode number used to link the participant to the conjunctival swab tube, the DBS card and consent form. A field inventory with the sample number and child’s name was also used. The 5-digit number was entered manually twice into the Android phone by the recorder for each study participant.

The survey team returned to the village in the evening to assess any participants who were absent during the initial examination.

### Laboratory procedures

Conjunctival swabs were tested for *C. trachomatis* via real-time PCR using the *C. trachomatis/Neisseria gonorrhoeae* (Ct/Ng) dual assay [23] in a COBAS 6800 (Roche Diagnostics) [24] which utilises an automatic onboard process from extraction to analysis. Testing was performed according to standard operating procedures and the manufacturer’s instructions [26]. Qualitative results were classified as per manufacturer’s instructions [24]. Only *C. trachomatis* results were evaluated for this study.

DBS were tested for anti-Pgp3 antibodies using ELISA and LFA [16,25]. For ELISA, optical density was measured at 450nm on a Sunrise plate reader (Tecan Group Ltd, Switzerland). The average result from 6 blank wells was subtracted from each averaged absorbance value, and the result normalised against the mid-range positive control to account for plate-to-plate variation [25]. A finite mixture model was used to classify the samples as seropositive or seronegative based on normalised absorbance values. The threshold for seropositivity was 0.249, determined by taking the mean of the Gaussian distribution of the seronegative population plus four standard deviations. For LFA, conjugate and capture reagents were prepared and dispensed onto a nitrocellulose membrane, with Pgp3 conjugated to InnovaCoat Gold (Innova Biosciences). The LFA device was constructed by placing components, such as absorbent pads, conjugate pads and membranes onto a backing card, cutting the assembly into strips and storing them at room temperature in a desiccator with low humidity [16].

Laboratory staff were masked to all clinical findings. Samples were de-identified prior to being sent to the laboratory.

Rigorous measures to prevent and identify contamination and for quality control were employed in the laboratory, including the following. Separate and distinct areas were maintained in the laboratory for sample preparation, and PCR amplification. Samples were uncapped in a class II biological safety hood, placed in racks with the collection swab remaining inside the tube, then directly transferred to the COBAS 6800 instrument. Gloves were changed between handling test reagents and samples. The assay reagents include uracil-n-glycoslyase, which prevents carry-over contamination within the instrument. We included AmpErase enzyme in the PCR master mix, which eliminates any contaminating amplicon from the previous PCR cycle, during the first subsequent thermal cycling step. DBS were tested in duplicate. For ELISA a blank spot was punched after each individual specimen spot to reduce the risk of carry-over contamination, and the CDC’s “Instructions for Use” quality control criteria were used for each testing plate. Samples and known-seronegative and known-seropositive human sera were tested for anti-Pgp3 antibodies on each plate. Plates were acceptable if results for 3 of 4 positive controls and the negative human serum control fell within pre-determined ranges (established during assay validation) and the positive controls were at least 50 times higher than background. Any plates that did not meet quality control criteria provided by the CDC were re-punched, re-eluted and re-tested. Each repeat plate passed quality control criteria.

### Statistical analysis

The TD dataset containing demographic, clinical and WASH variables was merged with the laboratory results dataset. The following descriptive analyses were undertaken: calculation of prevalence of TF, PCR positivity, and antibody positivity as well as proportions of each WASH variable; by dividing positive cases by overall examined/analysed respectively and adjusting for age and cluster which is the standard GTMP protocol for the analysis of TF and WASH data. For analysis purposes, water source variables were categorised as improved or unimproved as per the WHO/UNICEF Joint Monitoring Programme [26]. Household-level variables were attributed to each member of the household for mixed-effects logistic regression analysis described below.

A mixed-effects logistic regression analysis was used to assess the independent association of each potential explanatory variable (age, gender, WASH) with the presence of TF, PCR positivity (current infection), and antibody positivity (past infection). Models were adjusted for clustering at village level. Variables with a p-value of less than 0.25 in the univariable analysis were considered for inclusion in the multivariable analysis. The magnitude of association between the different variables and presence of TF, current infection and past infection was measured using crude odds ratios (cORs) and adjusted odds ratios (aORs) with 95% confidence intervals (CI). Analyses were undertaken in Stata v17 (StataCorp, College Station, TX, USA).

### Ethical considerations

This project was approved by the Solomon Islands Ministry of Health Ethics Committee (approval number HRE003_20), and the University of New South Wales (approval number HC190442). TD support for the surveys was approved by the London School of Hygiene & Tropical Medicine Observational Ethics Committee (16105).

## Results

### Study design and study participants

**Fig 1:**
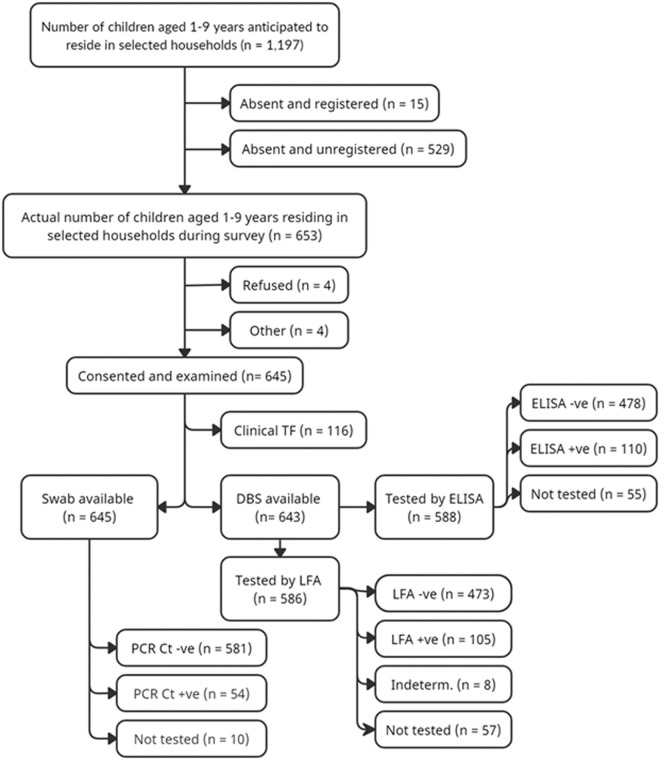
Summary of study recruitment, participants, and results. * Please see text for further information regarding excluded samples.

A total of 273 households across 15 villages were visited, in which 653 children aged 1–9 years were present and invited to participate in the study (Fig 1 and S2 Table). Of these, 4 children refused involvement with the study, and researchers were unable to examine 4 other children, while two children refused the DBS collection component. Overall, 645 children were examined and had conjunctival swabs collected, and 643 DBS were collected (Fig 1 and S2 Table).

During data collection, many houses were unoccupied as residents had travelled, including an estimated 529 children aged 1–9 years. No information was captured about the households or residents who were out of the village at the time of the study. Household WASH data were obtained for the 256 households in which children were examined. PCR analysis was carried out on swabs from 635 children; ELISA and LFA analysis was carried out on DBS from 588 and 586 children respectively. Initially, 2 of 17 plates (12%) did not meet quality control criteria provided by the CDC. These plates were re-punched, re-eluted and re-tested. Ten conjunctival swabs were excluded due to tube mislabelling. The 55 DBS samples excluded were deemed invalid due to heat exposure or damage from water, alcohol or lotion. A further 2 samples were unable to undergo LFA testing due to insufficient sample volume.

### Household WASH access

Almost 70% of households did not have access to an improved water source for washing with 63% of households using surface water for personal hygiene practices. In >60% of households, residents had to travel more than 30 minutes to collect water to wash, and only 3.3% had a water source piped into the household property to use for hygiene purposes (Table 1).

**Table 1:** Household water, sanitation and hygiene (WASH)* access (N = 273 households).

	Number (%)	95% CI (%)
**Water variables**		
**Washing water source**		
Improved water source	33 (12.1)	4 - 32
Unimproved water source	240 (87.9)	68 - 96
**Time to collect washing water**		
Water in yard	68 (24.9)	9 - 54
<30 mins	32 (11.7)	4 - 29
30–60 mins	97 (35.5)	17 - 60
>1 hr	76 (27.8)	9 - 60
**Sanitation variable**		
**Access to latrine**		
Yes	6 (2.2)	0.9 - 5.4
No	267 (97.8)	94 - 99

* The full list of WASH variables is presented in S3 Table.

Only 2.2% of households had access to improved sanitation, with 97.8% of households reporting that adults performed outdoor defecation. Only 4 households reported having their own latrine, of which 3 had a handwashing facility within 15 metres of the latrine supplied with water and soap (Tables 1 and S3).

### Prevalence of TF, conjunctival *C. trachomatis* infection, and anti-Pgp3 antibodies

**Fig 2:**
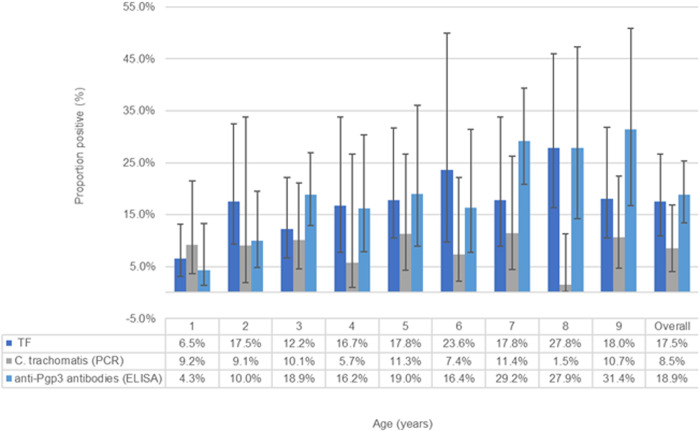
Prevalence of trachomatous inflammation—follicular (TF), conjunctival *Chlamydia trachomatis* (by PCR), and anti-Pgp3 antibodies detected by ELISA. Whiskers represent 95% CIs.

The overall prevalence of TF in the sample of children aged 1–9 years was 17.5% (95% CI 11–27) (Table 2). The proportion of children with TF in one or both eyes increased with age, with the lowest prevalence in children aged 1 year at 6.5%, increasing to 27.8% in children aged 8 years (Fig 2).

**Table 2:** Presence of trachomatous inflammation—follicular (TF) and conjunctival *Chlamydia trachomatis* in children aged 1-9 years, age-adjusted, Choiseul, 2020.

	n	Positive n	Positive %	95% CI (%)
TF	672	116	17.5	11 - 27
*C. trachomatis*	652	56	8.5	4 - 17
Anti-Pgp3 antibodies	602	113	18.9	14 - 28

Kappa =0.098, SE 0.019; 95% CI -0.135 to 0.060 (between TF and *C. trachomatis*).

A similar age pattern was seen with the ELISA results (evidence of previous infection with *C. trachomatis*) where 4.3% of children aged 1 year were positive for anti-Pgp3 antibodies by ELISA, increasing to 31.4% in children aged 9 years (Fig 2). The overall prevalence of positive anti-Pgp3 antibodies by ELISA results in children aged 1–9 years was 18.9% (95% CI 14–28).

No such age-related pattern was seen with the PCR results (evidence of current *C. trachomatis* infection). The proportion of PCR-positive results ranged from 1.5% in children aged 8 years to 11.4% in children aged 7 years, and an overall PCR-positivity prevalence of 8.5% (95% CI 4–17) (Table 2 and Fig 2).

Of children with TF, only 17% (20/116) also tested positive for *C. trachomatis* (Supplementary Table 4), and 36% (40/112) tested positive for anti-Pgp3 antibodies (S5 Table). Of the children who tested positive for *C. trachomatis,* 50% (25/50) also tested positive for anti-Pgp3 antibodies (S6 Table). TF was found in all villages surveyed; however, *C. trachomatis* was detected in only 7 of 15 villages surveyed, with one cluster of villages (cluster 125, in which 16% of 1–9-year-olds had TF) returning no *C. trachomatis*-positive conjunctival swabs*.* Only one village did not have evidence of past infection (S2 Table).

### Factors associated with TF, and current and previous *C. trachomatis* infection

We examined the association between age, gender, and WASH access with the presence of TF in one or both eyes, a positive PCR result for *C. trachomatis* and a positive ELISA result for anti-Pgp3-antibodies in children aged 1–9 years. Increasing age was strongly associated with increased odds of TF (OR = 1.2 for each 1-year increase, p < 0.001), however no other variable including gender or WASH variables showed an association with the presence of TF (Table 3). None of the factors tested in the univariable models had a p-value <0.25 for *C. trachomatis* infection and therefore we did not construct a multivariable model (S7 Table). Increasing age was strongly associated with increased odds of the existence of anti-Pgp3 antibodies (OR = 1.35 for each 1-year increase, p < 0.001) (S8 Table).

**Table 3:** Univariable and multivariable models examining the association between having TF in one or both eyes, age, gender, and WASH variables, in children aged 1–9 years.

Variable		n	TF (%)	Univariable model OR^*^ (95% CI†); p-value	Multivariable model
OR (95% CI); p-value
Age, increase per 1 year		668		1.2 (1.11–1.38); < 0.001	1.2 (1.11–1.38); < 0.001
Gender	Male	333	62 (18.6)	Reference	Reference
	Female	335	54 (16.1)	0.84 (0.50–1.41); 0.509	0.83 (0.49–1.42); 0.505
WASH					
Household water source for washing	Unimproved	608	108 (17.8)	Reference	Reference
	Improved	64	8 (12.5)	0.86 (0.23–3.25); 0.828	0.92 (0.22–3.8); 0.911
Access to a latrine	No	658	1 (7.1)	Reference	Reference
	Yes	14	115 (17.5)	0.35 (0.24–5.26); 0.451	0.32 (0.18–5.44); 0.428

* Odds ratio.

†Confidence interval.

Neither gender nor water source (improved versus unimproved) showed evidence of being associated with any of the three outcome variables. The evidence also did not support there being an association between access to a latrine and TF or anti-Pgp3 antibodies. It was not possible to test for an association with *C. trachomatis* due to the small number of households with a positive conjunctival swab with latrines.

## Discussion

This study aimed to investigate signs of trachoma in Choiseul and asses the relationship with previous or current *C. trachomatis* infection. The study was initiated in response to an unexpected TF result of 10.6% in Choiseul’s 2019 pre-validation surveillance survey. Our primary objectives were to establish whether the follicular conjunctivitis observed in Choiseul could be attributed to *C. trachomatis* and to provide data to establish whether trachoma is likely to be a public health problem in Choiseul requiring resumption of MDA.

We found a TF prevalence of 17.5% and *C. trachomatis* DNA in 8.5% of conjunctival swabs. These findings suggest that the previous 2019 pre-validation surveillance survey results are attributable to C. *trachomatis*, and that trachoma is a public health problem in this population, supporting the WHO recommendations for MDA in Choiseul. Three rounds of MDA are planned, with the first having occurred in September 2024. Given that TT prevalence in adults in 2019 was 0%, perhaps it is worth considering including investigations of early scarring by looking at limbal disease for future studies, as has been undertaken elsewhere in the Pacific Islands [27].

On the other hand, of individuals with TF, 83% had no evidence of current *C. trachomatis* infection, and 64% were negative for anti-Pgp3 antibodies for past exposure to *C. trachomatis,* suggesting that a proportion of TF in Choiseul is likely to be attributable to factors other than *C. trachomatis*. This conclusion aligns with findings from other Pacific Island countries [7,10,20,28,29], however, the organism(s) driving follicular conjunctivitis in the absence of *C. trachomatis* in this region have not been identified. Regardless of cause, the potential long-term impact of inflammation for affected individuals is unknown.

Among the children who tested positive for *C. trachomatis* by PCR (indicating current infection), 63% did not have TF. These cases may have had early infection, prior to the development of follicles, or a low infection load.

Although previous studies have established that poor WASH conditions are associated with greater likelihood of TF [30,31], our analysis did not identify a significant association between these factors and TF, *C. trachomatis* infection, or evidence of prior infection. This may reflect contextual differences in the Solomon Islands compared to high prevalence settings sub-Saharan Africa, where most of the existing evidence originates. For example, hygiene behaviour in arid African regions is strongly influenced by water scarcity [32], however regions in the Solomon Islands experience high rainfall and extreme weather events such as flooding that compromises clean water and sanitation facilities [33]. Whilst both regions practice open defecation, in arid African regions faeces is left exposed available for flies to breed [34], whereas in coastal Pacific communities, open defecation into the sea may limit fly breeding. These behavioural and environmental contexts may explain the absence of association in our analysis, and contribute to the unusual relationship observed between TF, *C. trachomatis*, Pgp3 seropositivity, and TT in this region. Nevertheless, both our analysis in Choiseul and previous studies in Western Province [35] show that the population of Solomon Islands is facing persistent challenges to secure improved water sources and sanitation facilities. Modelling suggests that the threshold for breaking the transmission of trachoma is a community access to sanitation of over 80% [30]. There is an urgent need for infrastructure improvements, which could reduce not only trachoma transmission but also the burden of other infectious diseases.

Our analysis also revealed that increasing age was strongly associated with increased odds of both TF and prior, but not current, *C. trachomatis* infection. The increase in seropositivity with age was expected given the long persistence of IgG antibodies in peripheral blood [14]. The increase in TF with age in our study is atypical: in most trachoma-endemic populations, pre-school-aged children are the main reservoir of infection and are most likely to have active trachoma. However a similar pattern has been observed in other Pacific Island countries, including Nauru [4,29].

A limitation of this study was the high number of unenrolled children, with an estimated 529 resident children absent at the time of the survey. However, it is possible that the number of resident children was overestimated, as the mean number of children per household used for this calculation was the national mean and may not reflect local demographic patterns. Furthermore, this area experiences a high level of movement to and from other Solomon Islands provinces and neighbouring Papua New Guinea, which could potentially explain relatively rapid changes in TF prevalence. We suggest that impact surveys conducted post-MDA also include investigations on the mobility of the population. A minor limitation of our work is the number of samples that could not be tested. Given these comprised under 10% of total samples collected, we do not believe this significantly affected the results. Conducting field-based research in the Pacific Islands is challenging for a variety of reasons including logistics, rapidly changing weather conditions and humidity. Strategies for improving field procedures, informed by the lessons learned from this exercise, will enhance procedures for future similar work.

In conclusion, while decisions regarding trachoma MDA have usually relied on TF prevalence, which until recently was the only feasible option in the field for assessing trachoma, it is now possible, as illustrated here, to integrate testing for current and previous *C. trachomatis* infection. This is of particular interest in settings like Choiseul where TF prevalence bounced back when MDA was stopped and there was the need to confirm whether current infection levels justified resuming MDA.

Acknowledgments The authors thank the staff of the Solomon Islands Ministry of Health and Medical Services, the team members that undertook data collection and the residents of Choiseul who participated in this study. The authors alone are responsible for the views expressed in this article and they do not necessarily represent the views, decisions or policies of the institutions with which they are affiliated.

## Supporting information

S1 Table Presence of trachomatous inflammation—follicular (TF) in children aged 1–9 years, age-adjusted, Choiseul, 2019. (DOCX)

S2 Table Number and proportion of children aged 1–9 years with positive results for trachomatous inflammation—follicular (TF), polymerase chain reaction (PCR), enzyme-linked immunosorbent assay (ELISA) and lateral flow assay (LFA). (DOCX)

S3 Table Complete Household Water, sanitation and hygiene (WASH) access (N = 273 households). (DOCX)

S4 Table Comparison of Positive and Negative Results for TF and current *C. trachomatis* Infection. (DOCX)

S5 Table Comparison of Positive and Negative Results for TF and Anti-Pgp3 antibodies. (DOCX)

S6 Table Comparison of Positive and Negative Results for *C. trachomatis* Infection and Anti-Pgp3 antibodies. (DOCX)

S7 Table Univariable model examining the association between the presence of Chlamydia trachomatis (CT), and age, gender, and WASH variables, in children aged 1–9 years. (DOCX)

S8 Table Univariable and multivariable models examining the association between the presence of anti-PGP3 antibodies (via ELISA), and age, gender, and Water, sanitation and hygiene (WASH) variables, in children aged 1–9 years. (DOCX)
