# Supplementary material for: The prevalence of trachoma, ocular Chlamydia trachomatis infection and anti-Pgp3 antibodies in Choiseul Province, Solomon Islands
Source: PLoS Negl Trop Dis. 2025 Sep 8;19(9):e0013381. doi: 10.1371/journal.pntd.0013381 (PMC12425259; doi:10.1371/journal.pntd.0013381)
Supplement: S1 Table — (DOCX) [file pntd.0013381.s001.docx]

|  | n | Positive n | Positive % | 95% CI (%) |
| --- | --- | --- | --- | --- |
| TF | 1046 | 125 | 10.64 | 6.9–14.4 |
