# Supplementary material for: The prevalence of trachoma, ocular Chlamydia trachomatis infection and anti-Pgp3 antibodies in Choiseul Province, Solomon Islands
Source: PLoS Negl Trop Dis. 2025 Sep 8;19(9):e0013381. doi: 10.1371/journal.pntd.0013381 (PMC12425259; doi:10.1371/journal.pntd.0013381)
Supplement: S2 Table — (DOCX) [file pntd.0013381.s002.docx]

**Supplementary Table 1. Number and proportion of children aged 1-9 years with positive results for trachomatous inflammation—follicular (TF), polymerase chain reaction (PCR), enzyme-linked immunosorbent assay (ELISA) and lateral flow assay (LFA)**

|  |  |  | **2019 Survey** | **2020 Survey** | | | |
| --- | --- | --- | --- | --- | --- | --- | --- |
| **Village Status** | **Population Total** | **Estimated pop (1-9 years)** | **TF +ve/Children examined (%)** | **TF +ve/Children examined (%)** | **PCR +ve/Samples tested (%)** | **Elisa +ve/Samples tested (%)** | **LFA +ve/Samples tested (%)** |
| Index Village 101 | 788 | 236 | 17/48 (35) | 25/64 (39.1) | 9/64 (14.1) | 18/63(28.6) | 17/62 (27.4) |
| Neighbouring Village 101a | 506 | 152 |  | 8/37 (21.6) | 6/37 (16.2) | 10/37 (27) | 11/37 (29.7) |
| Neighbouring Village 101b | 889 | 267 |  | 21/46 (45.7) | 14/46 (30.4) | 17/46 (37) | 19/46 (41.3) |
| Index Village 126 | 219 | 66 | 9/34 (26) | 1 /18 (5.6) | 1/18 (5.6) | 0/13 (0) | 0/13 (0) |
| Neighbouring Village 126a | 127 | 38 |  | 2/24 (8.3) | 0/24 (0) | 2/22 (9.1) | 2/22 (9) |
| Neighbouring Village 126b | 135 | 41 |  | 6/43 (14) | 10/42 (23.8) | 14/42 (33.3) | 13/47 (27.7) |
| Index Village 125 | 188 | 56 | 8/33 (24) | 4/16 (25) | 0/16 (0) | 2/16 (12.5) | 1/16 (6.3) |
| Neighbouring Village 125a | 99 | 30 |  | 5/42 (11.9) | 0/41 (0) | 2/37 (5.4) | 1/37 (2.7) |
| Neighbouring Village 125b | 218 | 65 |  | 7/42 (16.7) | 0/42 (0) | 6/42 (14.3) | 4/42 (9.5) |
| Index Village 123 | 155 | 47 | 17/34 (50) | 11/94 (11.7) | 0/91 (0) | 13/89 (14.6) | 14/89 (15.7) |
| Neighbouring Village 123a | 77 | 23 |  | 2/26 (7.7) | 1/25 (4) | 8/25 (32) | 7/17 (4.1) |
| Neighbouring Village 123b | 188 | 56 |  | 14/53 (26.4) | 0/52 (0) | 6/43 (12.8) | 6/43 (14) |
| Index Village 122 | 268 | 80 | 9/40 (22.5) | 7/95 (7.4) | 13/94 (13.8) | 1/20 (13.9) | 9/72 (12.5) |
| Neighbouring Village 122a | 46 | 14 |  | 1/23 (4.3) | 0/21 (0) | 1/20 (5) | 0/20 (0) |
| Neighbouring Village 122b | 88 | 26 |  | 2/22(9.1) | 0/21 (0) | 1/19 (5.2) | 1/19 (5.2) |
| **Total** | **3991** | **1197** | **60/189 (32)** | **116/645 (18)** | **54/635 (8.5)** | **110/588 (18.7)** | **105/586 (17.9)** |
