# Supplementary material for: The prevalence of trachoma, ocular Chlamydia trachomatis infection and anti-Pgp3 antibodies in Choiseul Province, Solomon Islands
Source: PLoS Negl Trop Dis. 2025 Sep 8;19(9):e0013381. doi: 10.1371/journal.pntd.0013381 (PMC12425259; doi:10.1371/journal.pntd.0013381)
Supplement: S3 Table — (DOCX) [file pntd.0013381.s003.docx]

**Supplementary Table 2: Complete Household Water, sanitation and hygiene (WASH) access (N=273 households)**

| Variables | **Number (%)** | 95% CI (%) |
| --- | --- | --- |
| **Water variables** | |  |
| **Drinking water source*** |  |  |
| Piped into yard | 55 (**20.1**) | 16.7 – 22.7 |
| Public tap/standpipe | 26 (**9.5**) | 6.2 – 10.4 |
| Unprotected dug well | 40 (**14.7**) | 13.6 – 19.2 |
| Unprotected spring | 15 (**5.5**) | 4.0 – 7.5 |
| Rainwater | 2 (**0.7**) | 0.2 – 1.6 |
| Surface water | 134 (**49.1**) | 46.2 – 53.8 |
| Other | 1 (**0.3**) | 0.0 -1.1 |
| Improved water source | 83 (**30.4**) |  |
| **Time to collect drinking water*** |  |  |
| Water in yard | 70 (**25.6**) | 9.1 -54.4 |
| <30 mins | 31 (**11.4**) | 4.1 – 27.9 |
| 30–60 mins | 94 (**34.4**) | 16.1 – 58.9 |
| >1 hr | 78 (**28.6**) | 9.4 – 60.8 |
| **Washing water source*** |  |  |
| Piped into yard | 9 (**3.3**) | 0.9 – 11.6 |
| Public tap/standpipe | 23 (**8.4**) | 2 – 28.9 |
| Protected dug well | 1 (**0.4**) | 0 – 3 |
| Unprotected dug well | 48 (**17.6**) | 4.6 – 48.4 |
| Unprotected spring | 18 (**6.6**) | 1.3 – 27.1 |
| Surface water | 173 (**63.4**) | 38.6 – 82.7 |
| Other | 1 (**0.4**) | 0 – 3.2 |
| Improved water source | 33 (**12.1**) |  |
| **Time to collect washing water**** |  |  |
| Water in yard | 68 (**24.9**) | 8.6 – 53.9 |
| <30 mins | 32 (**11.7**) | 4.2 – 28.6 |
| 30–60 mins | 97 (**35.5**) | 16.9 – 59.9 |
| >1 hr | 76 (**27.8**) | 9.1 – 59.8 |
| **Sanitation variables** | |  |
| **Where adults usually defecate** |  |  |
| Shared/public latrine | 2 (**0.7**) | 0.1 – 6 |
| Private latrine | 4 (**1.5**) | 0.6 – 3.8 |
| No structure/outside | 267 (**97.8**) | 37.3 – 84.7 |
| **Sanitation facility type at home** |  |  |
| F/P to septic tank | 1 (**0.4**) | 0.1 – 2.6 |
| F/P to pit latrine | 4 (**1.5**) | 0.4 – 5.3 |
| F/P to open drains | 1 (**0.4**) | 0 – 3.2 |
| No facilities/open defaecation | 267 (**97.8**) | 94.2 – 98.9 |
| Improved sanitation | 6 (**2.2**) | 0 – 3.2 |
| **Child faeces disposal method**** |  |  |
| Put in drain/ditch | 1 (**0.4**) | 0.1 – 2.6 |
| Buried | 2 (**0.7**) | 0.2 – 3.4 |
| Left in the open | 43 (**15.8**) | 7.4 – 30.3 |
| Other | 123 (**45.1**) | 28.3 – 63 |
| N/A (no child <3) | 104 (**38.1**) | 25.9 - 52 |
| **Hygiene variables** | |  |
| **Handwashing facility 15 m from latrine?*** |  |  |
| Yes | 3 (**1.1**) | 0.4 – 3.3 |
| No | 268 (**98.9**) | 96.7 – 99.6 |
| **Water at handwashing facility?** |  |  |
| Yes | 3 (**100**) |  |
| No | 0 |  |
| **Soap/ash at handwashing facility?** |  |  |
| Yes | 3 (**100**) |  |
| No | 0 |  |
