# Supplementary material for: The prevalence of trachoma, ocular Chlamydia trachomatis infection and anti-Pgp3 antibodies in Choiseul Province, Solomon Islands
Source: PLoS Negl Trop Dis. 2025 Sep 8;19(9):e0013381. doi: 10.1371/journal.pntd.0013381 (PMC12425259; doi:10.1371/journal.pntd.0013381)
Supplement: S4 Table — (DOCX) [file pntd.0013381.s004.docx]

**Supplementary Table 3. Comparison of Positive and Negative Results for TF and current *C. trachomatis* Infection**

|  | TF ^-ve^ | TF ^+ve^ | Total |
| --- | --- | --- | --- |
| CT ^-ve^ | 485 | 96 | 581 |
| CT ^+ve^ | 34 | 20 | 54 |
| Total | 519 | 116 | 635 |
