# Supplementary material for: The prevalence of trachoma, ocular Chlamydia trachomatis infection and anti-Pgp3 antibodies in Choiseul Province, Solomon Islands
Source: PLoS Negl Trop Dis. 2025 Sep 8;19(9):e0013381. doi: 10.1371/journal.pntd.0013381 (PMC12425259; doi:10.1371/journal.pntd.0013381)
Supplement: S5 Table — (DOCX) [file pntd.0013381.s005.docx]

**Supplementary Table 4. Comparison of Positive and Negative Results for TF and Anti-Pgp3 antibodies.**

|  | TF ^-ve^ | TF ^+ve^ | Total |
| --- | --- | --- | --- |
| Anti-Pgp3 ^-ve^ | 406 | 72 | 478 |
| Anti-Pgp3 ^+ve^ | 70 | 40 | 110 |
| Total | 476 | 112 | 588 |
