# Supplementary material for: The prevalence of trachoma, ocular Chlamydia trachomatis infection and anti-Pgp3 antibodies in Choiseul Province, Solomon Islands
Source: PLoS Negl Trop Dis. 2025 Sep 8;19(9):e0013381. doi: 10.1371/journal.pntd.0013381 (PMC12425259; doi:10.1371/journal.pntd.0013381)
Supplement: S6 Table — (DOCX) [file pntd.0013381.s006.docx]

**Supplementary Table 5. Comparison of Positive and Negative Results for current C. *trachomatis* Infection and Anti-Pgp3 antibodies.**

|  | CT -ve | CT +ve | Total |
| --- | --- | --- | --- |
| Anti-Pgp3 ^-ve^ | 464 | 25 | 489 |
| Anti-Pgp3 ^+ve^ | 88 | 25 | 113 |
| Total | 552 | 50 | 602 |
