# Supplementary material for: The prevalence of trachoma, ocular Chlamydia trachomatis infection and anti-Pgp3 antibodies in Choiseul Province, Solomon Islands
Source: PLoS Negl Trop Dis. 2025 Sep 8;19(9):e0013381. doi: 10.1371/journal.pntd.0013381 (PMC12425259; doi:10.1371/journal.pntd.0013381)
Supplement: S7 Table — (DOCX) [file pntd.0013381.s007.docx]

**Supplementary Table 6. Univariable model examining the association between the presence of *Chlamydia trachomatis* (CT), and age, gender, and WASH variables, in children aged 1–9 years.**

| Variable | n | *CT* ^+ve^ (%) | Univariable model  OR (95% CI); p-value |  |
| --- | --- | --- | --- | --- |
|  |  |  |  |  |
| Age, increase per 1 year | 635 | 8.5 | 1.1 (0.88-1.27); 0.561 |  |
| Gender |  |  |  |  |
| Male | 311 | 27 (8.7) | Reference |  |
| Female | 324 | 27 (8.3) | 0.88 (0.35-2.22); 0.797 |  |
| WASH |  |  |  |  |
| Household water source for washing |  |  |  |  |
| Unimproved | 527 | 50 (8.7) | Reference |  |
| Improved | 58 | 4 (6.9) | 0.89 (0.002-4.21); 0.219 |  |
|  |  |  |  |  |
| Access to a latrine |  |  |  |  |
| No | 624 | 54 (8.7) | Reference |  |
| Yes | 11 | 0 |  |  |
|  |  |  |  |  |
