# Supplementary material for: The prevalence of trachoma, ocular Chlamydia trachomatis infection and anti-Pgp3 antibodies in Choiseul Province, Solomon Islands
Source: PLoS Negl Trop Dis. 2025 Sep 8;19(9):e0013381. doi: 10.1371/journal.pntd.0013381 (PMC12425259; doi:10.1371/journal.pntd.0013381)
Supplement: S8 Table — (DOCX) [file pntd.0013381.s008.docx]

**Supplementary Table 7. Univariable and multivariable models examining the association between the presence of anti-PGP3 antibodies (via ELISA), and age, gender, and Water, sanitation and hygiene (WASH) variables, in children aged 1–9 years.**

| Variable | n | *Anit-PGP3* ^+ve^ *(*%*)* | Univariable model OR (95% CI); p-value | Multivariable model |
| --- | --- | --- | --- | --- |
|  |  |  |  | OR (95% CI); p-value |
| Age, increase per 1 year | 588 | 18.8 | 1.35 (1.19-1.52); <0.001 | 1.35 (1.19-1.53); <0.001 |
| Gender |  |  |  |  |
| Male | 284 | 52 (18.3) | Reference | Reference |
| Female | 304 | 58 (19.1) | 1.02 (0.61-1.7); 0.942 | 1 (0.57-1.74); 0.998 |
| WASH |  |  |  |  |
| Household water source for washing |  |  |  |  |
| Unimproved | 534 | 101 (18.9) | Reference | Reference |
| Improved | 54 | 9 (16.7) | 0.62 (0.17-2.28); 0.475 | 0.63 (0.14-2.44); 0.465 |
|  |  |  |  |  |
| Access to a latrine |  |  |  |  |
| No | 578 | 109 (18.9) | Reference | Reference |
| Yes | 10 | 1 (10) | 0.53 (0.04 - 8.04); 0.655 | 0.63 (0.03-11.93); 0.761 |
|  |  |  |  |  |
